# Acknowledgment of Reviewers

**DOI:** 10.1128/mBio.00195-16

**Published:** 2016-03-08

**Authors:** Arturo Casadevall

## EDITORIAL

On behalf of the editors of *mBio*, I gratefully acknowledge the following individuals, who served as reviewers for the journal in 2015. Their time and effort in reviewing articles have been essential in ensuring the high quality of our publications, and their help is greatly appreciated.

Mark Achtman

Margaret Ackerman

Alvaro Acosta-Serrano

Jeffrey Actor

Mark Adams

Pia Adelroth

Sarah Ades

Hector Aguilar

Brian Ahmer

Gillian Air

Klaus Aktories

Christopher Alabi

Sonja-Verena Albers

Courtney Aldrich

Caitilyn Allen

Francis Alonzo

J. Andrew Alspaugh

Lisa Alvarez-Cohen

James Alwine

Zhiqiang An

Deborah Anderson

Kirk E. Anderson

E. Virginia Armbrust

Sandra Armstrong

Gustavo Arrizabalaga

Michel Arthur

David Askew

Ina Atrée

Taeok Bae

Fabio Bagnoli

Joel Baines

Mona Bajaj-Elliot

David Baker

Nathalie Balaban

Ashwin Balagopal

Mitchell Balish

Emily Balskus

David Baltrus

Jeri Barak

Montserrat Barcena

Daniela Barilla

Carolina Barillas-Mury

Jeremy Barr

Rodolphe Barrangou

Christopher Basler

Andreas Bäumler

Kenneth Bayles

Pascale Beauregard

Avraham Beer

Peter Beernink

Robert Belas

John Belisle

Stephen Bell

Gabriele Berg

Jeffrey Bergelson

David Bermudes

Thomas Bernhardt

Sukesh Bhaumik

Jennifer F. Biddle

Paulo Bispo

Ira Blader

David Blair

Steven Blanke

Bonnie Blomberg

David Bloom

Linda Bockenstedt

Teun Boekhout

Blaise Boles

Robert Bonomo

Elizabeth Boon

Seth Bordenstein

Tanja Bosak

Jürgen Bosch

Helena Boshoff

Jonathan Boyson

Alexander Bradley

Peter Bradley

Thomas Briese

Brandon Briggs

David Briles

Paul Brindley

Eric Brown

Gordon Brown

Jeremy Brown

Glenn Browning

John Brumell

Brian Brunelle

Mark Brynildsen

Juliane Bubeck-Wardenburg

Kerry Buchholz

Angus Buckling

Andries Budding

Lori Burrows

David Bzik

Javier Cabanes

Isabelle Caldelari

Leonides Calvo-Bado

Andrew Camilli

Elizabeth Campbell

Andrea Campisano

Paula Cannon

Hannah Carey

Mark Carrington

Clayton Caswell

Prabha Chandrasekaran

Christophe Chassard

Keith Chater

Subhadeep Chatterjee

Dhruba Chattoraj

Hualan Chen

Jiandong Chen

Joseph Chen

Yury Chernoff

Ambrose Cheung

Scott Chimileski

Charles Chiu

Byung-Kwan Cho

Claire Chougnet

Peter Christie

Gino Cingolani

Anthony Clark

Thomas Clavel

Keith Clay

David Coil

Anamaris Colberg-Poley

Stephane Compant

Laurie Comstock

Sean Conlan

Nancy Connell

Isabelle Coppens

Richard Cordaux

Otto Cordero

Pierre Cornelis

Teresa Coque

Peggy Cotter

Harry Courtney

Patrice Courvalin

Leah Cowen

Michael Cox

Nancy Cox

Carolyn Coyne

Robert Cramer

Sean Crosson

Longzhu Cui

Melanie Cushion

Mauro Dalla Serra

Pranav Danthi

Dana Davis

Piet de Groot

René De Mot

Carlos de Noronha

Willem de Vos

Kirk Deitsch

Frank DeLeo

Maurizio Del Poeta

Neal DeLuca

Mark Denison

William DePas

Vojo Deretic

Andrew Desbois

Kevin Devine

Patricia Diaz

Andreas Diepold

Stephen Diggle

Larry Dishaw

Maziar Divangahi

Yohei Doi

Anca Dorhoi

Adam Driks

Patrick Duffy

Paul Dunman

Julie Dunning Hotopp

Gary Dunny

Magdalena Dunowska

Rebecca Dutch

Bas Dutilh

Jonathan Dworkin

Dan Dwyer

Michelle Dzeijman

Ashlee Earl

Frank Ebel

Leo Eberl

Tassos Economou

Andrew Edwards

Maryna Eichelberger

Patrick Eichenberger

Bart Eijkelkamp

Abraham Eisenstark

David Engelthaler

Luis Enjuanes

Lynn Enquist

Suzanne Epstein

Joel Ernst

Robert Ernst

Jeffery Errington

Joana Falcao Salles

Daniel Falush

Michael Federle

Brice Felden

Marta Feldmesser

Mariet Feltkamp

Gen-Sheng Feng

Hanping Feng

Peter Feng

Scott Ferrenberg

Paul Fey

Raina Fichorova

Paul Fidel

David Fidock

Ken Fields

Luisa Figueiredo

Melanie Filiatrault

Scott Filler

Alain Filloux

Ralf-Joerg Fischer

Vincent Fischetti

Matthew Fisher

Derek Fisher

Ross Fitzgerald

Barry Flinn

Harry Flint

Viacheslav Fofanov

Juan Fontana

Anja Forche

Larry Forney

Naomi Forrester

Kristoffer Forslund

Ron Fouchier

Carolin Frank

Richard Frankel

Michael Franklin

Lori Frappier

James Fraser

David Fredricks

Christopher French

W. Florian Fricke

David Friedman

Takema Fukatsu

Shinji Fukuda

Clay Fuqua

Tom Gallagher

J. Victor Garcia

Dominique Garcin

Timothy Garrett

Elke Genersch

Stephane Genin

Nicole Gerardo

Kenn Gerdes

Karine Gibbs

Jack Gilbert

Scott Gilbert

Michael Gillings

Zemer Gitai

Pierre Gladieux

Anna Gogleva

Hana Golding

Mark Gomelsky

Félix Goñi

Carlos Gonzalez

Uri Gophna

Christian Gortázar

Ernest Gould

Jeffrey Gralnick

Lone Gram

Gregor Grass

Mark Gresnigt

Joel Griffitts

Marc-Jan Gubbels

Patricia Guerry-Kopecko

Hubertus Haas

Ted Hackstadt

Julius Clemence Hafalla

Daisuke Hagiwara

Anders Hakansson

Mohamed Hakimi

William Hanage

Lynn Hancock

Hermie Harmsen

Simon Harris

Tajie Harris

Elizabeth Harry

Rasika Harshey

Elizabeth Hartland

John Harty

Kim Hasenkrug

Graham Hatfull

Philippe Hauser

Stanley Hazen

Cynthia He

Ian Head

Sophie Helaine

Nicholas Heng

Scott Hensley

Juan Hermoso

Alfredo Herrera-Estrella

Johannes Herrmann

Penelope Higgs

Hubert Hilbi

Russell Hill

James Hoch

Ann Hochschild

Robert M. Hoffman

Monica Höfte

Deborah Hogan

Brenda Hogue

Edward J. Hollox

Edward Holmes

Kathryn Holmes

Fred Homa

Hee-Jeon Hong

Marcus Horn

Alexander Horswill

Benjamin A. Horwitz

S. H. R. Hosseini

Benjamin Howden

Guanghua Huang

Jean Huang

Julie Huber

Casey Hubert

David Hunstad

Julian Hurdle

Aeron Hurt

Curtis Huttenhower

Alexander Idnurm

James Imlay

William Inskeep

Vikas Jain

Guilhem Janbon

Yu-Sin Jang

Blythe Janowiak

Gunther Jansen

Laura Jarboe

Michael Jennings

Lars Jeuken

Christian Jobin

Paul Johnston

Kristina Jonas

Jeffrey Jones

Aras Kadioglu

Jiri Kana

Hyun Ah Kang

Petros Karakousis

Kazuyuki Kasahara

David Katz

Deepak Kaushal

Barbara Kazmierczak

Daniel Kearns

Patrick Keeling

Melissa Kendall

François Képès

Thomas Kepler

Margaret Kielian

Mogens Kilian

Kami Kim

Samantha King

Veljo Kisand

Daan Kiviet

Lisa Klasson

David Knipe

Laura Knoll

Dennis Ko

Lester Kobzik

Andrew Koh

Heidi Kong

Omry Koren

Anita Koshy

Akos T. Kovacs

Natacha Kremer

Thomas Kristie

James Kronstad

Lee Kroos

Tomoko Kubori

Meta Kuehn

Michael Kühl

Jens Kuhn

Richard Kuhn

Purnima Kumar

Rolf Kümmerli

Andrei Kuzminov

Kyung Kwon-Chung

Peter Kwong

Marc-Andre Lachance

Michael Lagunoff

Thomas Lane

Jean-Paul Latge

Beth Lazazzera

Francois Lebreton

Benhur Lee

Elissa Lei

Jay Lennon

Alan Leonard

Emil Lesho

Elena Levashina

Bruce Levin

Tera Levin

Jesica Levingston Macleod

Stuart Levitz

Deborah Lewinsohn

Kim Lewis

Daniel Libraty

Tami Lieberman

Susan Liebman

Patrick Linder

Frederique Lisacek

Melissa Lodoen

Frank Loeffler

Richard Longnecker

Daniel López

Stephen Lory

Anice Lowen

Steven Lower

Alexander Loy

Shan Lu

Aldons Lusis

Michael Lynch

Susan Lynch

Bing Ma

Li-Jun Ma

Hiten Madhani

Kira Makarova

Mark Mandel

Riccardo Manganelli

Suliana Manley

Jaan Mannik

Costas Maranas

Esperanza Martínez-Romero

Flávia Martinello

Vega Masignani

Revati Masilamani

Sergei Maslov

Tetsuro Matano

Anuja Mathew

Ichiro Matsumura

Kai Matuschewski

Wendy Maury

Robin May

Shonna McBride

Christopher McDevitt

Diane McDougald

Michael McInerney

James McInerney

James McKinlay

Rachel McLoughlin

Barry McMahon

Paul L. McNeil

Espen Melum

Tod Merkel

D. Merrell

Dennis Metzger

Folker Meyer

Jan Michiels

Tam Mignot

Virginia Miller

David Mills

Aaron Mitchell

Yorgo Modis

Christopher Mody

Daniel Monleón

Donald Morisson

Cécile Morlot

Joachim Morschhäuser

Herbert Morse

Eric Mortensen

Rosa Mourino

W. Moye-Rowley

Conrad Mullineaux

Alicia Muro-Pastor

Barbara Murray

Lieve Naesens

Julian Naglik

Xavier Nassif

Sergei Nekhai

David Nelson

Josh Neufeld

David Newburg

Mari-Anne Newman

Olivier Neyrolles

David Nichols

Anthony Nicola

Kirsten Nielsen

Michele Nishiguchi

Joshua Nosanchuk

Mairi Noverr

Minou Nowrousian

Jack Nunberg

Takuro Nunoura

Marco Rinaldo Oggioni

Dennis Ohman

Antonio Oliver

Michal Olszewski

Carlos Orihuela

Alice Ortmann

Mario Ostrowski

Paul O’Toole

Melanie Ott

Karen Ottemann

Michael Otto

Soufian Ouchan

Kelli Palmer

Bernhard Palsson

Timothy Palzkill

Eric Pamer

Navraj Pannu

Leslie Parent

Adam Parks

Matthew Parsek

Norman Pavelka

Vladimir Pelicic

Philip Pellett

John Perfect

David Perlin

Stanley Perlman

Jean Luc Pernodet

Reuben Peters

Kevin Pethe

Constantinos Petrovas

Julie Pfeiffer

Andrew Phipps

Dominique Pidard

Susan Pierce

Robert Pike

Mariana Pinho

Paul Planet

Julie Plastino

Joe Pogliano

Stefan Pöhlmann

Shawn Polson

Martin Polz

Mihai Pop

Danilo Porro

Jennifer Potts

Lance Price

Morgan Price

David Pride

James Prosser

Paolo Puccetti

Ralf Rabus

Seth Rakoff-Nahoum

Sanjay Ram

Richard Randall

Philip Rather

Daniel Read

Timothy Read

Matthew Redinbo

Christopher Rensing

Federico Rey

Lisa Reynolds

Todd Reynolds

Andrew Rice

Kelly Rice

Anthony Richardson

Cristina Risco Ortiz

Jeffrey Roberts

Marilyn Roberts

Eduardo Rocha

Dan Rockey

Marcio Rodrigues

John Rohde

Antonis Rokas

Suzan Rooijakkers

R. Roop

Jason Rosch

Ilan Rosenshine

Natividad Ruiz

Steffen Rupp

Charles Russell

Jacob Russell

Kathleen Ryan

Jonah Sacha

Jeroen Saeij

Jason Sahl

Padmini Salgame

Jeanne Salje

Nicole Sampson

Scott Samuels

Alvaro Sanchez

Per Saris

Christopher Sassetti

Tor Savidge

Luis Sayavedra-Soto

Esperance Schaefer

Herb Schellhorn

Siegfried Scherer

Jeffrey Schertzer

Larry Schlesinger

Amy Schmid

Claudia Schmidt-Dannert

Christoph Schoen

Christoph Schüller

Martin Schuster

Martin Schwemmle

Patrick Seed

Anna Seekatz

Julia Segre

H. Seifert

Yuji Sekiguchi

David Sela

Bret Sellman

Bert Semler

Ganes Sen

Paolo Serafini

Angela Sessitch

Peter Setlow

Konstantin Severinov

William Shafer

Mara Shainheit

Sagi Shapira

Itai Sharon

Paul Sharp

Thomas Sharpton

Brian Shaw

Bang Shen

Bo Shopsin

D. V. Sign

Anita Sil

Thomas Silhavy

Natalie Silmon de Monerri

Lynn Silver

Robert Silverman

Eric Skaar

Mark Slifka

Jason Slot

Mark Smeltzer

C. Smith

Joe Smith

George Smulian

Evan Snitkin

Evgeni Sokurenko

Dominique Soldati-Favre

Abraham Sonenshein

Joseph Sorg

Brad Spellberg

Nicola Stanley-Wall

Jack Stapleton

George Stewart

Timothy Stinear

Ulrich Stingl

Gregory Storch

Klemen Strle

Jorg Stulke

Xin-zhuan Su

Carlos Subauste

Periasamy Subbarayan

William Sullivan

Bryan Swingle

W. Swords

Anna Székely

Hendrik Szurmant

Yi-Wei Tang

Phil Tarr

Jeffery Taubenberger

John Taylor

Tanel Tenson

Rita Tewari

Kevin Theis

Ulrich Theopold

Alex Therien

Casey Theriot

Volker Thiel

Marco Thines

Paul Thomas

Torsten Thomas

Vinai Thomas

Linda Thomashow

Justin Thornton

J. Cameron Thrash

Alejandro Toledo-Arana

Eva Top

Alfredo Torres

Victor Torres

Baldwyn Torto

Guy Tran Van Nhieu

Matthew Traxler

Moritz Treeck

Elaine Tuomanen

Peter Turnbaugh

Jeffery Twiss

Buddy Ullman

Gottfried Unden

Constantin Urban

Raphael Valdivia

Susana Valente

Peter van der Ley

Gerlinde van de Walle

Giel van Dooren

Laurence Van Melderen

Mark van Raaij

Willem van Schaik

Daria Van Tyne

Rachel Vannette

Willem van Wamel

Raghavan Varadarajan

Michael Vasil

Vijayakumar Velu

Keerthi Prasad Venkataramanan

Salvador Ventura

James Versalovic

Kevin Verstrepen

Laura Via

Marco Vignuzzi

Waldemar Vollmer

Veronika von Messling

Andreas Wack

Stephen Waggoner

Matthew Waldor

K. Walker

Judy Wall

David Wang

Dong Wang

Jin-Town Wang

Digby Warner

Christopher Waters

Milind Watve

Joel Weadge

Gunter Wegener

Grzegorz Wegrzyn

Jeffrey Weiser

David Weiss

Matthew Welch

Sandra Weller

Melanie Wellington

Finn Werner

Guido Werner

John Werren

Judith White

Marvin Whiteley

Chris Whitfield

William Whitman

Brian Wilkinson

Alex Wilson

Malcolm Winkler

Wade Winkler

Sebastian Winter

Elizabeth Winzeler

Jeffrey Withey

Steven Witkin

Christiane Wolz

Daniel Wozniak

Jianming Wu

Marcel Wüthrich

Karina Xavier

Jie Xiao

Jin-Rong Xu

X. Frank Yang

Robert Yarchoan

Lael Yonker

Sung Yoon

Lingchong You

Vincent Young

Xi Yu

Andrew Yurochko

Eva Yus Najera

Nicholas Zachos

Thomas Zahrt

Oscar Zaragoza

Yanjin Zhang

Yifan Zhang

Bojian Zheng

Wilma Ziebuhr

Joseph Ziegelbauer

